# Oral Sensory Sensitivity Influences Attentional Bias to Food Logo Images in Children: A Preliminary Investigation

**DOI:** 10.3389/fpsyg.2022.895516

**Published:** 2022-06-22

**Authors:** Anna Wallisch, Lauren M. Little, Amanda S. Bruce, Brenda Salley

**Affiliations:** ^1^Juniper Gardens Children’s Project, University of Kansas, Kansas City, KS, United States; ^2^Department of Occupational Therapy, Rush University, Chicago, IL, United States; ^3^Department of Pediatrics, University of Kansas Medical Center, Kansas City, KS, United States; ^4^Center for Children’s Healthy Lifestyles and Nutrition, Kansas City, MO, United States

**Keywords:** food advertising, sensory processing, picky eating, eye tracking, attentional bias

## Abstract

**Background:**

Children’s sensory processing patterns are linked with their eating habits; children with increased sensory sensitivity are often picky eaters. Research suggests that children’s eating habits are also partially influenced by attention to food and beverage advertising. However, the extent to which sensory processing influences children’s attention to food cues remains unknown. Therefore, we examined the attentional bias patterns to food vs. non-food logos among children 4–12 years with and without increased oral sensory sensitivity.

**Design:**

Children were categorized into high (*n* = 8) vs. typical (*n* = 36) oral sensory sensitivity by the Sensory Profile-2. We used eye-tracking to examine orientation and attentional bias to food vs. non-food logos among children with high vs. typical oral sensory sensitivity. We used a mixed model regression to test the influence of oral sensory sensitivity to attentional biases to food vs. non-food logos among children.

**Results:**

Results showed that children with high oral sensory sensitivity showed attentional biases toward non-food logos; specifically, children with high oral sensory sensitivity oriented more quickly to non-food logos as compared to food logos (*p* < 0.05), as well as spent more time looking at non-food logos as compared to food logos (*p* < 0.05). Findings were in the opposite direction for children with typical oral sensory sensitivity.

**Conclusion:**

Sensory sensitivity may be an individual characteristic that serves as a protective mechanism against susceptibility to food and beverage advertising in young children.

## Introduction

Evidence shows that children’s sensory processing patterns impact their eating habits, as children with sensory sensitivity are described as picky eaters and often refuse novel foods [for a review see Dunn et al. (2016)]. Extensive research also demonstrates that increased food and beverage advertising is positively associated with children’s consumption of unhealthy foods [for a review see Boyland et al. (2016)]. While investigators have examined the influence of individuals’ weight and age on differential patterns of attention to food advertising (e.g., Carters et al., 2015), research has not yet addressed how children’s sensory processing patterns may be associated with attentional bias to food advertisements. For children with sensory sensitivity, food advertisements may not be particularly rewarding, contributing to decreased attention to advertising cues. Therefore, this study examined the extent to which sensory processing sensitivity influenced children’s attentional bias to food logos. This is important because findings from this study may contribute to an understanding of how specific sensory processing patterns serve as a protective factor against the effects of unhealthy food and beverage advertising.

According to the *Reactivity to Embedded Food Cues in Advertising Model*, individual susceptibility factors influence physiological and psychological reactivity to food cues, which leads to a reciprocal relationship with eating behavior (i.e., incentive-sensitization process) (Folkvord et al., 2016). This model is supported by studies which show that children’s individual characteristics (e.g., impulsivity, attention) are related to their food choices. For example, Folkvord et al. (2014) showed that children with higher impulsivity were more susceptible to food marketing than children with lower impulsivity. Another study revealed that children who showed increased attention (e.g., gaze duration, number of fixations) to food cues were more likely to eat unhealthy snacks (Folkvord et al., 2015). Velazquez and Pasch (2014) found that children’s preferences for unhealthy food were associated with their maintenance of attention to food logos; however, associations were no longer significant after controlling for demographic characteristics. Lastly, Spielvogel et al. (2018) found that unhealthy food cues attracted children’s visual attention to a greater extent than healthy food cues, although, children’s initial visual interest (i.e., latency to first fixation) did not differ between unhealthy and healthy food cues. We propose that the way children experience food and marketing based on their sensory processing patterns may underlie attentional biases to food cues, and may serve as an individual factor that can help clarify the mixed findings related to children’s attention to food cues.

Emerging evidence suggests that sensory processing may be an individual characteristic that influences individuals’ attention to food cues and subsequent eating behavior. Sensory processing refers to the ways in which individuals detect and behaviorally respond to sensory information. According to Dunn’s Framework of Sensory Processing (Dunn, 2014), individuals demonstrate behaviors that reflect underlying neurological thresholds. Children that have high neurological thresholds require more intense or an increased amount of sensory stimulation to notice aspects of their environments (i.e., these children are considered *underresponsive*). Conversely, children that have low neurological thresholds notice environmental stimuli very quickly and can easily become overwhelmed by environmental stimuli (i.e., these children are *sensory sensitive*). Sensory processing patterns may differ across systems (e.g., tactile, auditory) and individuals may show both high and low neurological thresholds based on sensory system (Dunn, 2014).

Studies show that children with sensory sensitivity demonstrate greater physiological reactivity [for review see Aron et al. (2012)], while other literature suggests those with sensory sensitivity show highly selective eating patterns (e.g., Farrow and Coulthard, 2012). Conversely, children with high sensory thresholds (i.e., underresponsive) are more likely to be obese (e.g., Davis et al., 2013). Additional research shows that sensory sensitivity is associated with picky eating (Steinsbekk et al., 2017). However, many studies have linked overall sensory sensitivity and/or a combination of visual, auditory, and tactile stimuli with selective eating (e.g., Wildes et al., 2012; Nederkoorn et al., 2015). It is unclear if children’s sensory patterns, however, are related to their attention to environmental food cues; it may be that the reactivity among children with increased sensitivity results in negative experiences with food, which in turn contributes to overall decreased attention to food cues in the environment. In other words, the association between oral sensitivity and attention to food cues has not been investigated. In the current study, we investigated the following research question: To what extent does oral sensory sensitivity impact attention to food vs. non-food logo images among typically developing children ages 4–12 years old? We hypothesized that children with high oral sensory sensitivity would show different patterns of attention to food vs. non-food logos. Specifically, we hypothesized that children with high oral sensory sensitivity would demonstrate increased duration of attention to non-food logos as compared to those with typical oral sensory sensitivity.

## Materials and Methods

### Participants

We recruited 44 children ages 4–12 years through local community organizations. We obtained approval from the institutional review board at the University of Kansas Medical Center and children’s legal guardians provided informed consent. Children were excluded if they had a history of a developmental diagnosis, vision/hearing/physical impairments, uncontrolled seizure disorder, and/or history of traumatic brain injury. We also excluded children with a gastrointestinal condition (e.g., gastro-esophageal reflux, dysphagia). The sample included children aged 49–148 months (*M* = 93.95 months, SD = 26.27 months). The sample was 52.3% female, and the average body mass index (BMI) was 17.12 (SD = 2.46, range = 12.38–23.27).

### Procedures

#### Stimuli

We presented a paired preference paradigm with 31 slides; each slide showed 2 images on opposite corners of the screen of a food logo and a non-food logo. All images were based on a paradigm of logo image presentation (see Bruce et al., 2012), which showed familiar food and non-food logos to children. The logos that were included in the current study were child appropriate and culturally familiar (e.g., common fast food chains, popular television streaming networks). While the original Bruce et al. (2012) study validated 120 images, our team used 62 logos (31 food, 31 non-food) that had not been altered by companies or corporations since the original study and may be most familiar to young children. Please refer to the Bruce et al. (2012) study for a full description of how the images were validated and rated according to familiarity, valence, and arousal as well as a full list of the images.

The images were presented on a white background, which was split into four equal quadrants. Each quadrant measured 8 in × 6 in (20.32 cm × 15.24 cm), and was presented full-screen on a 16 in × 12 in (40.64 cm × 30.48 cm) monitor. The two images within each stimulus were matched for size; each picture was confined to a space of 4 in × 4 in (10.16 cm × 10.16 cm). Each picture was centered within its quadrant, leaving a 4-inch (10.16 cm) gap between each picture horizontally, and a 2-inch (5.08 cm) gap between each picture vertically.

#### Eye Tracking Research Technology System

We presented images on a 16 × 12 in. computer monitor. Responses were recorded using Applied Science Laboratory (ASL) E6 eye-tracking system, Model 504 (Applied Science Laboratories [ASL], 2008) with the GazeTracker interface program (Eye-Gaze Response Interface Computer Aid [ERICA], Inc, 2010) in a darkened interior room. The pan/tilt module, a component of the ASL system, uses near infrared technology to illuminate the eye and telephoto an image of the eye onto a camera. The E6 control unit then extracts the pupil and reflection of the light source on the cornea to compute gaze location at a 120 Hz sampling rate. Each child was seated in a hydraulic chair that was adjusted to the child’s eye height with the mid-point of the stimulus monitor (124.5 cm). We used a 5-point standard calibration in which dynamic cartoons were presented individually at each of the target points. Once accurate calibration was achieved, the experimental paradigm proceeded and we monitored calibration throughout the session. If calibration was inaccurate, we paused the testing session, recalibrated, and resumed the session.

### Data Extraction and Reduction

We used the GazeTracker interface program to extract variables of interest within each defined look zone (i.e., food logo, non-food logo). All data was transferred to excel and each trial by variable was extracted per participant. We calculated the below variables of interest for *each trial* within *each participant*.

#### Fixation Count

Fixation count is the number of times an individual stops to examine each stimulus; the minimum time of each stop was set to 0.250 s, which includes return fixations (i.e., the number of times an individual looks at a stimulus on a slide, then returns attention to that stimulus again).

#### Latency to First Fixation

This variable represents the duration (in seconds) from the start of a trial until the participant visually fixates on either image.

#### Number of Times in Zone

Each image fell within an outlined zone (food logo, non-food logo) and this variable represents the number of times that each participant showed fixation counts within each zone.

#### Percent Time Spent in Zone

This variable represents the percent of total look duration within each zone (food vs. non-food logo zones), relative to overall looking time.

### Measures

The *Sensory Profile-2* (SP-2; Dunn, 2014) is a standardized parent-report tool used to evaluate a child’s sensory processing patterns in the context of everyday life. It consists of 86 questions which are scored using a 5-point Likert Scale. Parents indicate the extent to which each item describes their child’s experience and/or functioning (almost always to almost never). A variety of summary scores are generated reflecting patterns in three domains: Sensory Modalities (auditory, visual, touch, movement, body position, oral), Behavior (attention, conduct, social-emotional), and Sensory Processing Pattern (registration, seeking, sensitivity, avoiding). In the oral processing domain, *n* = 5 items are categorized as “oral sensitivity” and *n* = 5 items are categorized as “oral seeking”; we used the mean of the 5 items within the oral sensitivity domain to create a mean score.

We calculated an oral sensitivity score based on our hypothesis that oral sensitivity would influence children’s eye gaze to food vs. non-food logo stimuli. According to norm-referenced data, children are categorized as “*much less than others/less than others*,” “*similar to others*,” or “*more than/much more than others*”; such categorizations help practitioners understand individual’s scores as they relate to peers and to determine if they meet cut-off scores to show clear sensory differences. When children show ‘much less/less than others’ scores, they scored at least 1 SD below the majority of peers and are showing *decreased* responses to sensory stimuli in that domain. When children show “more than/much more than others,” scored at least 1 SD above the majority of peers and they are showing *increased or exaggerated* responses in that sensory domain.

### Data Analysis

As the *Sensory Profile-*2 scoring was standardized in the general population, we categorized children’s oral sensitivity scores into “much less than others/less than others”; “similar to others”; or “more than/much more than others” based on normative data. For the oral sensitivity score, we considered any score equal to or above 2.4 as “*more than/much more than others*” because in the normative scoring, a mean score on the sensitivity overall score above 2.4 (on a 5 point likert scale) is considered as such [refer to Dunn (2014)]. We then used SAS 9.2 (SAS Institute, 2015) to analyze data and used hierarchical linear modeling, also referred to as mixed model regression, to test research questions. The repeated administration of stimuli presentation to each participant introduces dependence in the measurement of outcomes, as responses are nested within individuals (Raudenbush and Bryk, 2002). Therefore, the estimation of random effects accounts for such dependence. We tested four models with the following as dependent variables: (1) fixation count; (2) latency to first fixation; (3) number of times in zone; and (4) percent time spent in zone. We treated each trial as repeated measures within child; we included sensory group (more/much more than others, similar to others, less than others) and condition (food logo, non-food logo) as independent variables. We also tested the interaction between sensory group × condition. This analytic approach allowed us to test the extent to which children with high vs. typical oral sensory processing may orient and attend to food vs. non-food logo images.

## Results

### Main Effects

In the current sample, *n* = 8 children showed high (i.e., “much more/more than others”) and *n* = 36 children showed typical (i.e., “similar than others”) oral sensitivity processing scores. The mean oral sensitivity score for the high (i.e., “much more/more than others”) group was 3.88 (SD = 0.80), while the mean oral sensitivity score for the typical (i.e., “similar than others”) group was 2.09 (SD = 0.25). Using a *t*-test, results showed that the high oral sensitivity group significantly differed from the typical oral sensitivity group (*p* < 0.001).

We then used a *t*-test to examine whether there was a significant difference in the chronological age between those with high vs. typical oral sensory sensitivity. Results showed that those with high oral sensory sensitivity were younger (mean age = 77.63 months) than those with typical scores (mean age = 97.58 months), but this did not reach significance (*t* = 2.011[42], *p* = 0.051). While not significant, we still controlled for age group in all subsequent analyses, as research shows a positive association between age and visual attention in children (Dye and Bavelier, 2010). We also used a *t*-test to examine differences in BMI between groups; results showed no significant differences (*t* = −1.09 [41], *p* = 0.914).

**FIGURE 1:**
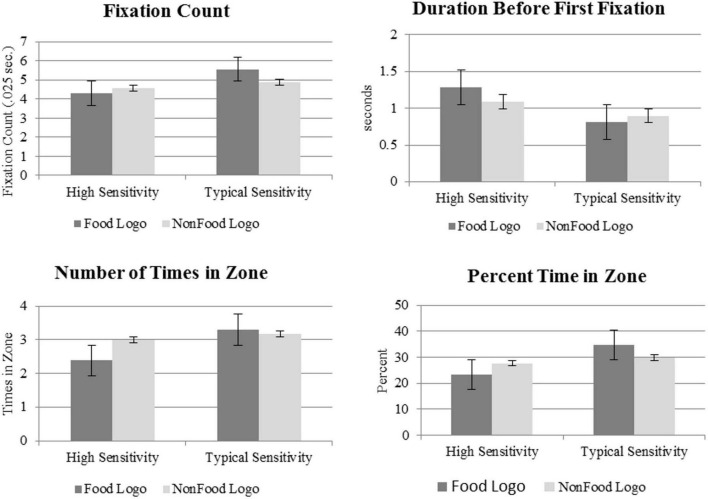
Mean scores on eye tracking variables of interest between the high oral sensitivity and typical oral sensitivity groups.

Significant main effects were not found; however, significant interactions between condition and group were found for fixation count (*p* < *0.05*), latency to first fixation (*p* < *0.01*), number of times in zone (*p* < *0.01*), and percent time spent in zone (*p* < *0.001*). See Table 1 for results. Given the significant main effects found for latency to first fixation, number of times in zone, and percent time spent in zone, we conducted follow up comparisons. See Figure 1 for mean scores across group.

**TABLE 1:** Type 3 tests of fixed effects.

			DF	*F* Value	*p*
**Fixation count**					
		Condition	1977	1.83	0.176
		Group	38.2	0.35	0.555
		Age_Group	38.6	0.16	0.688
		Condition × Group	1977	4.69	0.031
**Latency to first fixation**					
		Condition	2195	0.96	0.326
		Group	37.9	1.52	0.225
		Age_Group	38	0.03	0.864
		Condition × Group	2195	7.47	0.006
**Number of times in zone**					
		Condition	1973	3.62	0.057
		Group	34.4	2.82	0.102
		Age_Group	35.2	0.05	0.833
		Condition × Group	1973	7.31	0.007
**Percent time spent in zone**					
		Condition	2195	0.24	0.626
		Group	37.7	1.29	0.262
		Age_Group	37.7	0.11	0.745
		Condition × Group	2195	18.50	<0.0001

*Condition, food logo vs. non-food logo; group, oral sensory sensitivity high vs. oral sensory sensitivity typical.*

### Fixation Count

Children with typical oral sensory sensitivity showed a significantly higher fixation count for food vs. non-food logos (Estimate = 0.76, SE = 0.19, DF = 1984, *t*-value = 4.02, *p* < *0.0001*). Children with high oral sensory sensitivity did not show a significant difference in fixation count between stimuli type.

### Latency to First Fixation

Latency to first fixation refers to the amount of time before participants visually oriented to either the food or non-food logo stimuli. Mean scores showed that children with typical oral sensitivity first looked at food logo images in 0.81 s (SD = 0.97 s), vs. those with high oral sensitivity first looking at food within 1.28 s (SD = 1.19 s). Children with typical sensory oral processing oriented significantly more quickly to food (*M* = 0.81 s; SD = 0.97) vs. non-food logos (*M* = 0.90 s; SD = 0.97 s) (Estimate = −0.09, SE = 0.04, df = 2195, *t*-value = −2.08, *p* < *0.05*). Conversely, children with high oral sensitivity oriented significantly more quickly to non-food (*M* = 1.09; SD = 1.18 s) as compared to food logos (*M* = 1.28; SD = 1.19) (Estimate = 0.10, SE = 0.09, df = 195, *t*-value = 2.05, *p* < *0.05*).

### Number of Times in Zone

Children with typical oral sensitivity looked more to the food logo image zone (*M* = 3.36; SD = 2.65) compared to those with high oral sensitivity (*M* = 2.5; SD = 2.5) (Estimate = 1.04, SE = 0.44, DF = 47.1, *t*-value = 2.49, *p* < *0.05*). Children with typical oral sensory sensitivity showed no significant difference in the number of times in the food logo zone (*M* = 3.36; SD = 2.66) vs. non-food logo zone (*M* = 3.30; SD = 2.30) (Estimate = 0.12, SE = 0.14, *D* = 1970, *t*-value = 0.89, *p* = 0.37). However, children with high oral sensitivity looked more to the non-food zone (*M* = 3.28; SD = 3.13) vs. the food zone (*M* = 2.5; SD = 2.5) (Estimate = −0.71, SE = 0.28, *D* = 1969, *t*-value = −2.58, *p* < *0.01*).

### Percent Time Spent in Zone

Children with typical oral sensitivity spent a significantly higher percentage of looking time in the food zone (*M* = 34.71; SD = 23.45), compared to those with high oral sensitivity (*M* = 23.31; SD = 22.29) (Estimate = 9.87, SE = 4.49, df = 43.2, *t*-value = 2.20, *p* < *0.05*). Children with typical oral sensory processing spent a significantly higher percentage of time looking at food logos (*M* = 34.71; SD = 23.45) vs. non-food logo images (*M* = 29.78; SD = 21.29) (Estimate = 5.49, SE = 0.97, df = 2195, *t*-value = 5.68, *p* < 0.0001). However, children with high oral sensitivity spent a significantly greater percentage of looking time in the non-food zone (*M* = 27.69; SD = 25.18) vs. food (*M* = 23.31; SD = 22.29) (Estimate = −4.38, SE = 2.08, df = 2195, *t*-value = −2.10, *p* < *0.05*).

## Discussion

Findings from the current study suggest that oral sensory sensitivity influences children’s patterns of attention to food vs. non-food logo stimuli. Regardless of chronological age or BMI, results show that children with high oral sensory sensitivity display an orientation bias toward non-food logos and an overall attentional bias to non-food logos. Children with typical oral sensory sensitivity, however, show orientation and overall attentional biases toward food logos. Results from the current investigation show that oral sensory sensitivity may be a child characteristic that serves as a moderating factor in attentional bias to food advertising.

The current study’s findings highlight the important role of sensory sensitivity in children’s cue reactivity, which in turn may influence their eating habits. While high oral sensory sensitivity has been associated with picky eating (e.g., Nadon et al., 2011), it has previously been unclear if attention may play a pivotal role in this relationship. According to the *Reactivity to Embedded Food Cues in Advertising Model*, cue reactivity results in physiological responses to food cues in one’s environment (Folkvord et al., 2016). Children with high sensory sensitivity have been shown to have increased physiological arousal as compared to those with typical sensory sensitivity and/or sensory under-responsivity (e.g., Schaaf et al., 2010). Additionally, sensory sensitivity has been linked with increased sensitivity to disgust (Schienle and Schlintl, 2019). High oral sensory sensitivity may be a person characteristic that predisposes children to a negative physiological response to food cues, which then contributes to decreased attention to food cues. Taken together, our findings suggest that even in the absence of a diagnosed clinical eating disorder or difference in BMI, children with high oral sensory sensitivity show significant attentional differences to food advertising as compared to those with typical oral sensory sensitivity. While previous studies have uncovered the eating behavior differences among those with high sensory sensitivity, the current investigation points to the underlying role of attentional bias to non-food logos among those with such high sensory sensitivity. It may be that the motivation that drives attentional bias toward food in many children is not similar among those with increased oral sensory sensitivity.

This study provides novel information related to the link between attentional biases to food logos and oral sensitivity patterns. Specifically, children with higher oral sensory sensitivity demonstrated less attentional bias toward food logos; whereas, children with typical oral sensitivity patterns showed attentional biases toward food logos. This means that children with higher oral sensitivity patterns may show less motivation to look at images associated with food. Picky eating is often associated with higher oral sensitivity, and this means that picky eaters may attend less, and be less susceptible, to food and beverage advertising cues.

### Limitations and Future Directions

The limitations of the current study include our relatively modest sample size and a wide age range. Additionally, the paradigm that was used to elicit attention must be replicated and validated in a larger sample of children. Specifically, while our study utilized logos familiar to children and logos used in previous studies with children (e.g., Bruce et al., 2012; Boyland et al., 2016), certain logos may have been more or less arousing than others (e.g., toy logos vs. phone company logos). Thus, future studies may match food and non-food images based on the degree to which an image is exciting to children. Further, we did not capture the child’s level of hunger prior to viewing the eye tracking paradigms, and future studies should examine how hunger states may influence attentional biases. Children’s oral sensitivity may be associated with their overall sensitivity scores as well as other sub-domains (e.g., touch processing) that fall within the sensitivity score. In this study, we limited analyses to testing the influence of oral sensitivity on attention to logos. Lastly, we used a parent report measure of sensory processing, and while a validated measure, parent report may differ from individual child experiences. Future studies should include a larger sample with a narrower age range as well as include a behavioral measure of sensory processing.

## Data Availability Statement

The raw data supporting the conclusions of this article will be made available by the authors, without undue reservation.

## Ethics Statement

The studies involving human participants were reviewed and approved by University of Kansas Medical Center Institutional Review Board. Written informed consent to participate in this study was provided by the participants’ legal guardian/next of kin.

## Author Contributions

AW and LL designed the study, collected the data, analyzed the data, interpreted the data, and wrote this manuscript. AB and BS supported the design of the eye tracking paradigms, as well as the interpretation of findings. All authors contributed to this manuscript, reviewed drafts of the manuscript, provided critical feedback, and approved the final version.

## Conflict of Interest

The authors declare that the research was conducted in the absence of any commercial or financial relationships that could be construed as a potential conflict of interest.

## Publisher’s Note

All claims expressed in this article are solely those of the authors and do not necessarily represent those of their affiliated organizations, or those of the publisher, the editors and the reviewers. Any product that may be evaluated in this article, or claim that may be made by its manufacturer, is not guaranteed or endorsed by the publisher.
